# Very-low-birth-weight infants, prophylactic micafungin or fluconazole

**DOI:** 10.4103/0253-7613.71904

**Published:** 2010-12

**Authors:** Luis Gonzalez-Granado

**Affiliations:** Department of Pediatrics and Immunodeficiencies, Hospital 12 octubre, Carretera Andalucia km 5,400, Postal code 28041, Madrid, Spain. E-mail: nachgonzalez@gmail.com

Sir,

I read with great interest the article by Grover in a previous issue of the journal.[1] The author states that micafungin is approved for prophylaxis of candida infections in patients undergoing hematopoietic stem cell transplant (HSCT). It is not the only the use of prophylactic micafungin, but also it also prevents candida infection in very-low-birth-weight preterm neonates. The authors support the idea of micafungin as a second-line treatment after failure with fluconazole. However, both drugs have been assessed and the comparison in prophylaxis is favorable to micafungin.[2]

The pharmacokinetic profile of micafungin in very-low-birth-weight preterms has been studied, and its safety and efficacy in that population has been confirmed.[3] As it has been reported, the efficacy of micafungin in the clearance of candida infection compared with fluconazole is favorable to micafungin.[4] Therefore, we should recognize that micafungin is at least an alternative to fluconazole as the preferred drug for antifungal prophylaxis in very-low-birth-weight preterm patients.
